# Differentiating secondary from primary dengue using IgG to IgM ratio in early dengue: an observational hospital based clinico-serological study from North India

**DOI:** 10.1186/s12879-016-2053-6

**Published:** 2016-11-28

**Authors:** Khalid Hamid Changal, Ab Hameed Raina, Adnan Raina, Manzoor Raina, Rehana Bashir, Muzamil Latief, Tanveer Mir, Qayum Hamid Changal

**Affiliations:** 1 Mercy St. Vincent’s Medical Center, Toledo, 43608 Ohio USA; 2 Internal Medicine, Paras Super Specialty Hospital, Gurgaon, India; 3 Internal Medicine, Sher-i-Kashmir Institute of Medical Sciences, Srinagar, India; 4 Clinical Biochemistry, Sher-i-Kashmir Institute of Medical Sciences, Srinagar, India; 5 Institute of Dental Sciences and Technology, Chowdhary Charan Singh University, Modinagar, India; 6 Internal Medicine, Jamia Millia Islamia University, New Delhi, India; 7 University of Science and Technology, Chittagong, Bangladesh

**Keywords:** Dengue, Secondary dengue, ELISA, PCR

## Abstract

**Background:**

Secondary dengue causes more severe disease than the primary. Early on, it is important to differentiate the two. We tried to find important clinical and laboratory differences between the two for the purpose of early differentiation.

**Methods:**

One hundred fourteen patients confirmed on reverse transcriptase-polymerase chain reaction (RT PCR) were studied. On day 2 of illness IgM and IgG indices were studied for calculation of IgG/IgM ratio. A one-step immunochromatographic assay was used for classification of patients into primary and secondary dengue. Patient characteristics were also studied.

**Results:**

Dengue serotype 1 was the most common found in 60.5% patients. 66.7% (76 patients) had secondary dengue. Secondary dengue cases had a higher mean temperature (101.56 ± 1.55 vs. 100.79 ± 1.25,°F, p 0.015), lower platelet counts (50.51 ± 38.91 vs. 100.45 ± 38.66, x 10^3^/micl, *p* <0.0001) and a significantly higher percentage of Dengue hemorrhagic fever/Dengue shock syndrome (38.2% vs. 2.6%, *p* <0.0001). In early phase of dengue NS1 and PCR were found to be better tests for diagnosis and later IgM is better. The IgG/IgM ratio of ≥ 1.10 had a sensitivity of 100%, specificity of 97.4% and accuracy of 67.5% in differentiating secondary from primary dengue.

**Conclusion:**

Early on in the clinical course, IgG/ IgM ratio can play an important role to differentiate the two. We found the ratio of ≥ 1.10 to be the best cut off for the same.

## Background

### Summary

Dengue is a viral disease that is transmitted by mosquitoes in tropical areas. India is one of the countries maximally affected by it. Dengue can either cause a self limiting fever with low platelets or a severe disease characterized by capillary leak, intravascular volume depletion, severely low platelet counts and a high grade fever. There are 4 distinct types of dengue viruses known to cause dengue. Infection with a particular kind of dengue virus builds lifelong immunity to it, but the person is still vulnerable to be infected with the other types of dengue viruses. Infection with other kinds of dengue viruses in a person who already had a dengue fever in the past is called secondary dengue and has been found to be severe. This is due to more severe immunological response in the body [1]. In our study we found that secondary dengue caused more severe disease features like shock, low platelets and higher grade fever. We tried ELISA based tests to differentiate secondary dengue as they are cheaper and more easily available in resource poor countries which bear the brunt of the disease. IgG/IgM ratio of >1.10 is useful for diagnosis of secondary dengue early on in the illness when it is very difficult to predict which patients have secondary dengue using other features.

### Introduction

Dengue fever (DF) is a serious illness in many tropical and subtropical countries. In ancient Chinese literature it has been referred to as “water poison” due to its association with flying insects. The etiological organisms responsible are dengue viruses (DENV) belonging to the family Flaviviridae, genus *Flavivirus*. Dengue virus has 4 serotypes : 1,2,3,4. The WHO 2009 classification divides dengue depending on the severity of illness. It classifies dengue as with or without warning signs (pain abdomen, severe vomiting, fluid retention and third spacing, mucosal hemorrhage, malaise and drowsiness, hepatomegaly, high hematocrit with low platelets); and severe dengue (severe plasma leakage, severe bleeding, or organ failure) [2]. On the other hand 1997 classification divides it into undifferentiated fever, dengue fever (DF), and dengue haemorrhagic fever (DHF) [3]. We chose the latter definition in our study to allow for comparison with previous studies most of which have used the same definition. In India, Dengue has been known since 1946 and has been present in different parts of the country as epidemics and outbreaks. In 1960s it had established its endemicity in India and different strains have been replacing the older ones ever since [4]. Asymptomatic infections are much more frequent than symptomatic ones [5]. Severe DENV infection like dengue hemorrhagic fever (DHF) and dengue shock syndrome (DSS) are chiefly seen with secondary infection [6, 7]. Considering this, it becomes very important to distinguish primary from secondary dengue early on in the course of illness. It helps in predicting prognosis and also in deciding about whether a patient needs admission and close monitoring or could be managed at home. This becomes more important during epidemics and outbreaks when hospitals are flooded with patients and early triage becomes necessary.

The Hemagglutination inhibition (HI) test was the standard reference test recommended by the World Health Organization (WHO) to classify primary and secondary dengue virus infection [8]. This reference test requires paired serum samples and cannot give an early diagnosis, exhibits high cross-reactivity and requires chemical pre-treatment to remove nonspecific inhibitors of hemagglutination [9, 10]. So, researchers have tried to find out better and easier methods to differentiate primary from secondary dengue. IgG avidity can be used to discriminate among primary dengue and secondary dengue infection [11].

The aim of this study is to differentiate primary from secondary dengue using IgG to IgM ratio from enzyme-linked immunosorbent assays (ELISA) based detection of specific antibodies. The IgG and IgM ELISA has the benefit of easier use, being suitable for surveillance and for large-scale studies. Several studies have been performed to discriminate primary and secondary DENV infection using the ratio of IgG and IgM at the different days of onset of symptoms [12–16]. We look for the best IgG/IgM ratio to differentiate primary and secondary DENV infection in our population within the first three days of symptom onset.

## Methods

This study was conducted in two tertiary care hospitals in the National Capital Region of India. It was a prospective observational study done from July to December 2015 when the maximum numbers of dengue cases are recorded. Patients with suspected dengue fever who presented within 2 days of symptom onset were recruited in the study with proper informed consent. Two hundred twenty-five patients with suspected dengue were initially recruited in the study. Of these only those who tested positive for dengue on reverse transcriptase polymerase chain reaction (RT PCR) [Simplexa^TM^ Dengue RT-PCR, focus diagnostics] testing were studied further. Thus only 114 patients were studied further. Patients were studied for clinical, laboratory and serological patterns.

### Qualitative detection of NS-1, IgM and IgG

The SD BIOLINE Dengue Duo rapid test kit produced by Standard Diagnostics was used for classification of patients into primary and secondary dengue. It is a one-step immunochromatographic assay designed for the detection of both dengue virus NS1 antigen and differential IgM/IgG antibodies to dengue virus in whole blood, serum, or plasma. For SD BIOLINE dengue NS1 Ag device, 100 μL of the test sample was added into the sample well (S). Test results were interpreted at 15–20 min. Similarly, for SD BIOLINE dengue IgG/IgM device, 10 μL of test sample was added into the sample well (S). This was followed by the addition of 4 drops (90–120 μL) of assay diluent to the round shaped assay diluent well. Results were interpreted at 15–20 min. The test results were examined and interpreted according to the manufacturer instructions by three different readers to avoid biasness.

### Case definition of primary and secondary dengue

Primary infection was defined as an IgM-negative/IgG-negative; or IgM-positive/IgG-negative on the blood sample drawn within 3 days of symptom onset. Secondary infection was defined as an IgM-negative/IgG-positive or an IgM-positive/IgG-positive result on the blood sample drawn within 3 days of symptom onset. We could not differentiate secondary from tertiary or quarternary dengue so some patients classified as having secondary dengue could have had tertiary or quarternary dengue. Days of illness were calculated considering the day of onset of symptoms (like fever, body aches, vomiting etc.) as the first day. The sensitivity and specificity of the use of SD BIOLINE Dengue Duo rapid test kit to classify dengue cases into primary and secondary dengue infection is 94.2 and 96.4% respectively [17].

Another blood sample was drawn on day 7 after symptom onset for IgM to detect the rise in the IgM positive patients tested on the SD BIOLINE Dengue Duo rapid test kit.

### DENV IgM and IgG index measurement

The Focus Diagnostics Dengue Virus IgM and IgG Capture DxSelect^TM^ were used. Both are Indirect Enzyme-linked immunosorbent assay (ELISA) tests. The IgM assay uses a flavivirus group monoclonal horseradish peroxidase conjugate with TMB as substrate. Each Capture Well is coated with antihuman IgM. The antigen solution contains equal proportions of inactivated DV types 1–4. The polystyrene microwells are coated with anti-human antibody specific for IgM (μ -chain). Diluted serum samples and controls are incubated in the wells, and IgM present in the sample binds to the anti-human antibody (IgM specific) in the wells. Dengue virus (DV) antigen is then added to the wells and incubated; and, if anti-DV IgM is present in the sample, the DV antigen binds to the anti-DV in the well. Mouse anti-DV conjugated with horseradish peroxidase is then added to the wells and incubated. Enzyme substrate and chromogen are added, and the color is allowed to develop. After adding the Stop Reagent, the resultant color change is quantified by a spectrophotometric reading of optical density (OD) which is directly proportional to the amount of antigen-specific IgM present in the sample. Sample optical density readings are compared with reference cut-off OD readings to determine results. An index value of > 1.00 is presumptive for the presence of IgM antibodies to dengue virus.

IgG assay is also an indirect ELISA. The polystyrene microwells are coated with equal proportions of inactivated and purified Dengue virus types 1–4. Diluted serum samples and controls are incubated in the wells to allow specific antibody present in the samples to react with the antigen. Nonspecific reactants are removed by washing and peroxidase-conjugated anti-human IgG is added and reacts with specific IgG. Excess conjugate is removed by washing. Enzyme substrate and chromogen are added, and the color is allowed to develop. After adding the Stop Reagent, the resultant color change is quantified by a spectrophotometric reading whose principle is similar to the one described for IgM above. An index value of > 1.00 is presumptive for the presence of IgG antibodies to dengue virus.

### Ratio of IgG to IgM (IgG/IgM)

This was calculated by dividing the IgG index to the IgM index value for each sample. It is important to note that all the data related to this calculation was made on day 2 of the illness.

### Statistics

Statistical analysis was done on an MS Windows-based PC computer. The data were first keyed into a Microsoft Excel spreadsheet and then analyzed by Statistical Package for the Social Sciences (SPSS), Version 20.0, from SPSS incorporation Chicago IL. We used mean, standard deviation/standard error of mean, and percentage when appropriate for the patient’s characteristic description. Group differences were compared using the Pearson χ2 or Fisher’s exact test for categorical variables, or the Student t test or the Mann–Whitney U test for continuous variables. *P*-values of 0.05 or less were considered statistically significant. Sensitivity, specificity, accuracy and likelihood ratios were determined for different IgG/IgM ratios and the best value was determined by looking at the combination of these parameters. Receiver operator curves were determined for the chosen ratio and area under the curve (AUC) determined. An AUC of more than 0.9 is considered excellent.

### Ethics

The study was approved by Ethics approval boards of the hospitals where the study was conducted (Institute Ethics committee of Fortis Memorial Research Institute and Institute Ethics committee of Paras Super specialty Hospital). Written informed consent was obtained from all the patients involved in the study.

## Results

**Fig. 1:**
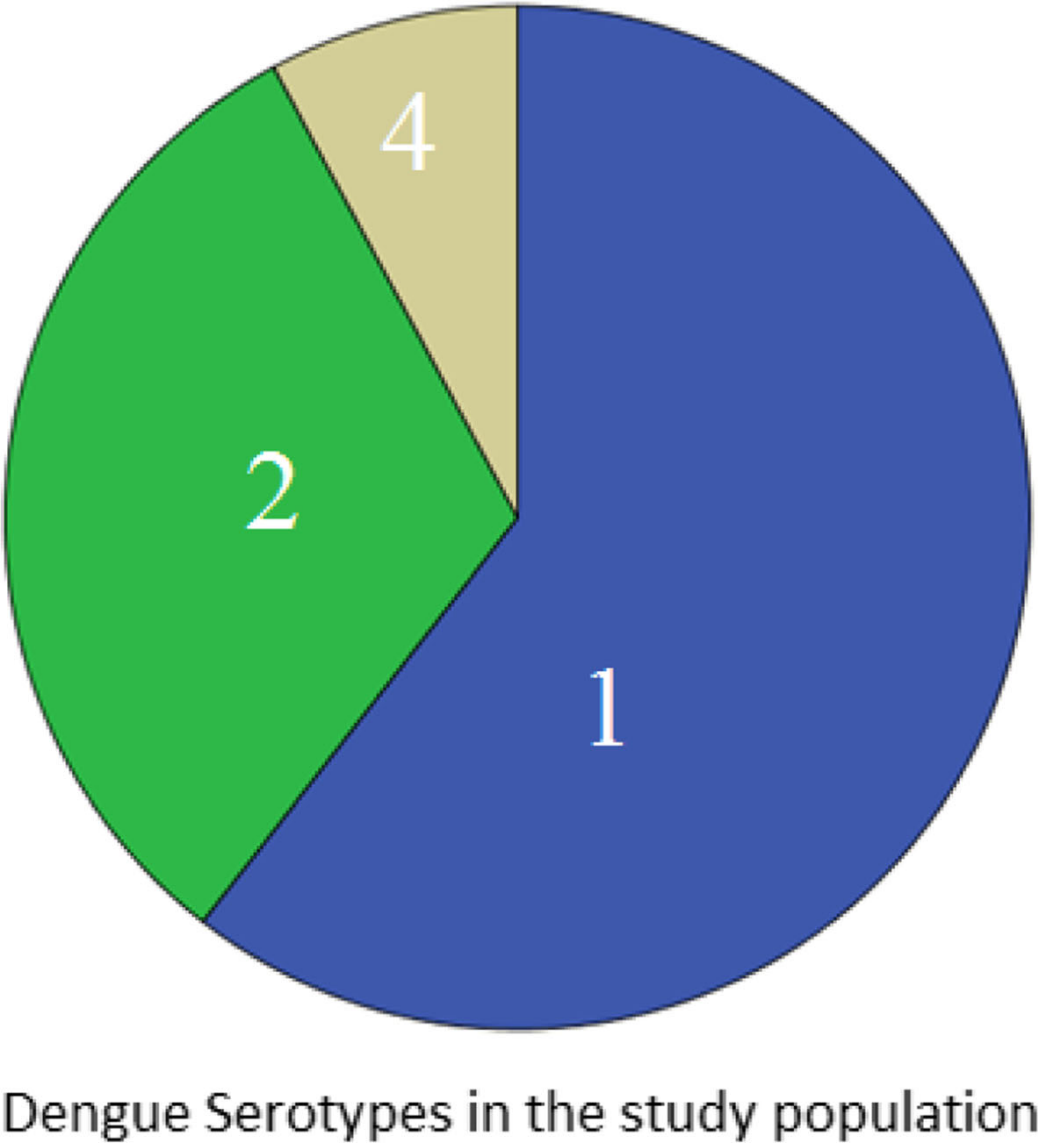
Pie Chart showing the frequencies of different Dengue virus serotypes found in our study. As can be seen Serotype 1 was the most common, found in 60.5% (*n* = 69), and followed by serotype 2 (31.6%, *n* = 36) and serotype 4 (7.9%, *n* = 8). Serotype 3 was not found in any patient

Of the 225 patients initially recruited 114 were confirmed by RT-PCR to be having dengue and were studied further. Dengue serotype 1 was the most common found in 60.5% patients (*n* = 69) (Fig. 1). 66.7% (76 patients) had secondary dengue and 33.3% (38 patients) had primary dengue. None of the patients were found to have serotype 3. Demographic characters, clinical features, laboratory data and serological features are shown in Table 1. Number of males is significantly higher than females. Secondary dengue patients’ have a lower mean age, higher mean maximum temperature, lower platelet counts, lower IgM index, higher IgG index, higher Ig G/ Ig M ratio and more chances of having DHF/DSS.

**Table 1:** Comparison of main characters of patients of primary and secondary dengue

Characteristic	Total patients	Primary dengue	Secondary dengue	*P* value
	*N* = 114	*N* = 38 (33.3%)	*N* = 76 (66.7%)	
Sex % (n)				
Males	70.2% (80)	76.3% (29)	67.1% (51)	0.387
Females	29.8% (34)	23.7% (9)	32.9% (25)	
Age (mean ± SD), Years	38.52 ± 10.99	42.18 ± 11.64	36.68 ± 10.24	0.011
Max temp. (mean ± SD), Fahrenheit	101.2 ± 1.51	100.79 ± 1.25	101.56 ± 1.558	0.015
Platelet count (mean ± SD) × 103/micl	67.16 ± 45.31	100.45 ± 38.66	50.51 ± 38.91	<0.0001
IgM index (mean ± SD)	1.21 ± 0.85	1.45 ± 1.24	1.09 ± 0.55	0.032
IgG index (mean ± SD)	2.81 ± 1.92	0.30 ± 0.14	4.06 ± 0.88	<0.0001
Ig M D2 % (N)	51.8% (59)	44.7% (17)	55.3% (42)	0.324
IgM D7 % (N)	96.5% (110)	97.4% (37)	96.1 (73)	1.0
IgG % (N)	66.7% (76)	0%	100% (76)	<0.0001
IgG/ IgM (mean ± SD)	3.22 ± 2.91	0.34 ± 0.27	4.65 ± 2.53	<0.0001
Dengue fever %(n)	73.7% (84)	97.4% (37)	61.8 (47)	<0.0001
DHF/DSS % (N)	26.3% (30)	2.6% (1)	38.2% (29)	
NS1 % (N)	90.4% (103)	92.1% (35)	89.5% (68)	0.749

This table shows the characteristics of the patients studied and compares amongst the primary and secondary cases

*Abbreviations*: *N* number, *SD* standard deviation, *DHF* dengue hemorrhagic fever, *DSS* dengue shock syndrome, *NS1* nonstructural protein 1 antigen, *Ig M D2* IgM done on day 2, *Ig M D7* IgM done on day 7

**Fig. 2:**
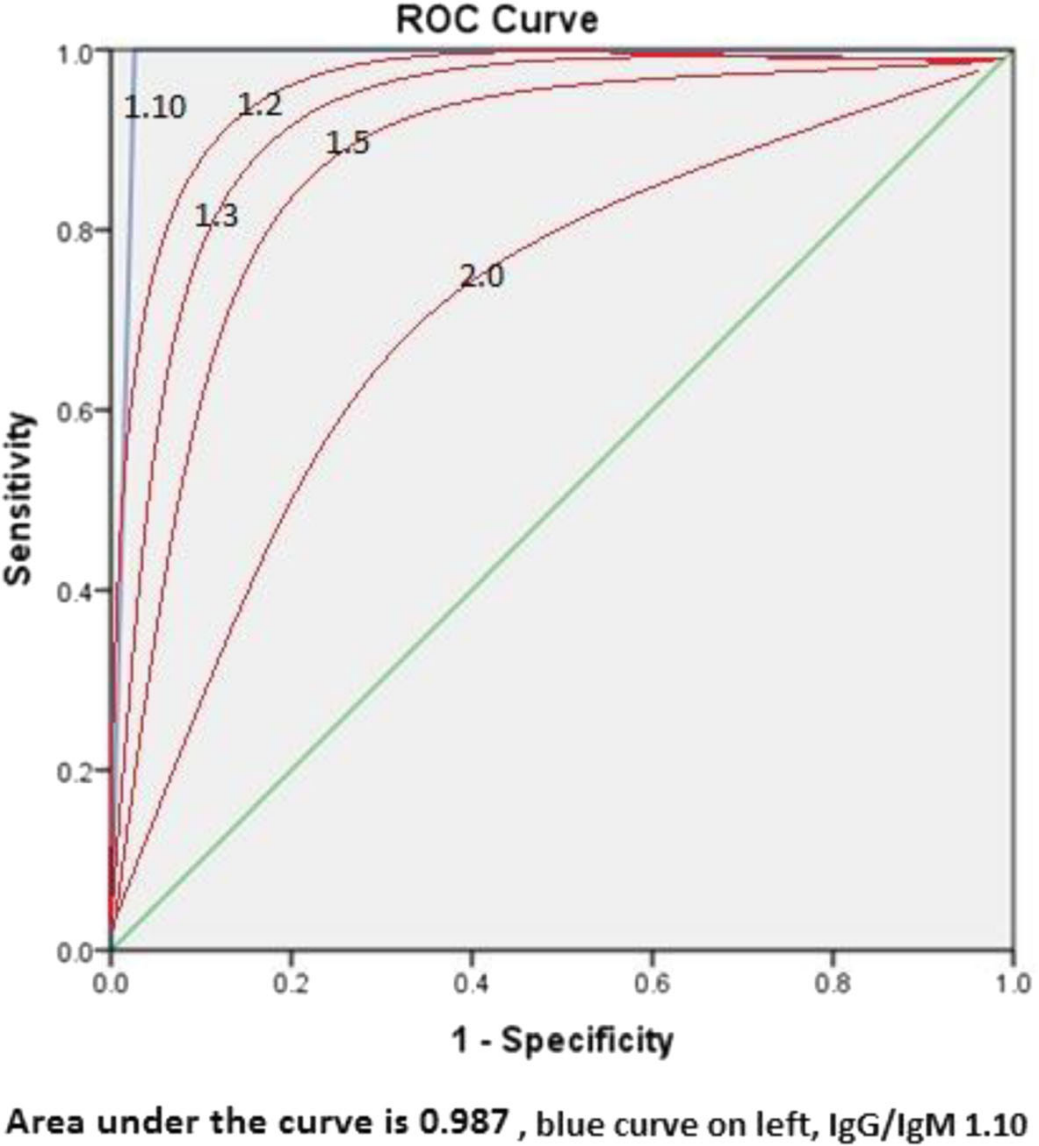
Receiver operator curve for IgG/IgM ratio of 1.10. The area under the curve is 0.987. The curve is on the left extreme and is in blue. The other red curves are for the IgG/IgM ratios of 1.2,1.3,1.5 and 2.0 respectively as we move to the diagonal line. 2.0 is closest to the diagonal line. The curve closest to the diagonal line is considered the least accurate. A value of more than 0.9 is considered excellent

Table 2 shows the comparison for different IgG/IgM ratios for the diagnosis of secondary dengue. A ratio of 1.10 was found to be the best for the diagnosis of secondary dengue with a sensitivity of 100%, specificity of 97.4% and a positive likelihood ratio of +38.46. Figure 2 shows the receiver operator curve (ROC) for the selected ratio to be 0.987 which is an excellent measure. The same figure also shows ROC for other ratios which were significantly lower.

**Table 2:** Different ratios of IgG/IgM for the diagnosis of secondary dengue fever

IgG/ IgM ratio	Sensitivity	Specificity	Accuracy	LR+	LR-
2.0	86.8	100%	57.9	∞	0.132
1.5	94%	100%	63.2	∞	0.06
1.4	94.7	100.0%	63.2	∞	0.053
1.3	94.7	100.0%	63.2	∞	0.053
1.2	97.4%	97.4%	65.8	37.4	0.026
1.15	100%	97.4%	67.5	+ 38.46	0
1.10^a^	100%	97.4%	67.5	+ 38.46	0
0.9	100%	65.8%	78.1%	2.9	0

This table shows the sensitivity, specificity, accuracy, LR+ and LR- calculated for different ratios of IgG/IgM for the diagnosis of secondary dengue fever

*Abbreviations*: *LR*+ positive likelihood ratio, *LR*- negative likelihood ratio

^a^Most useful value to diagnose secondary dengue. 1.10 is chosen as the most accurate ratio of IgG/IgM to diagnose based on all the variables used here

∞ value cannot be ascertained

## Discussion

The early diagnosis of secondary dengue is important because it has been recognized as a significant risk factor for development of severe dengue [13]. In our study, out of the 114 cases studied a majority was of secondary cases (66.7% vs. 33.3%). Other studies have also reported a higher number of secondary dengue cases [18, 19]. Higher number of secondary dengue infections occurs only in Dengue-endemic countries. Pathogenesis in dengue is linked to the host immune response, which is triggered by infection with the virus. Primary infection is usually benign. Secondary infection with a different serotype or multiple infections with different serotypes may, however, cause severe infection [20]. This leads secondary dengue to be clinically more apparent and thus more people seeking medical care. This is further evidenced in our study by secondary dengue cases having a higher mean maximum temperature, lower platelet counts and a significantly higher percentage of DHF/DSS. We had a much higher percentage of males in the study compared to the females (70.2% vs. 29.8%). A few other hospital based studies from India suggest a similar trend [21, 22]. This could be due to males spending more time outdoors in the conservative Indian society or due to differential seeking of medical care by different sexes or due to biological differences in the sexes to dengue virus. Serotype 1 was most common followed by 2 and 4. No DENV3 was isolated. Dengue virus type-1 was the prevalent serotype in another recently conducted study from North India [23]. Also we found that patients with secondary dengue were younger than those with primary dengue. Some other studies have reported otherwise [24].

IgM tested on day 2 was positive in 51.8% only while it increased to 96.5% on day 7 of the illness. This result reiterates the well accepted dictum that IgM ELISA is the test of choice after first 5 days of Illness [25]. NS1 is much easily detected in the first few days of dengue as was seen in 90.4% of our cases. Mean IgM index tested on day 2 of illness was found to be higher in primary cases than in secondary cases. On the other side, mean IgG index tested on day 2 was found to be almost 4 times higher in secondary dengue. Similar conclusions have been derived in some other studies too [12, 26, 27]. Thus in early phase of dengue NS1 and PCR are better tests for diagnosis and later IgM is better. This is true for both primary and secondary dengue fever.

IgG/ IgM ratio has been studied in many studies for early differentiation of primary and secondary dengue. Ratios done at different time stages of the disease have revealed different proposed cut offs [12, 14, 18, 28]. We found the IgG/ IgM ratio of 1.10 and higher to be the best cut off value for differentiating secondary from primary dengue. Ratios ranging from 1.1 to 2 have been proposed in other studies. These differing ratios can be due to difference in the times of collection of sera, differences in the populations being studied and differences in the laboratory methods studied. Cucunawangsih et al. conducted a study in Indonesia where the ratio was measured within 3 days of onset of disease and found the best ratio to be 1.14. Although their laboratory methods are very similar to ours but there sample size is almost three times lower than ours (48 vs. 114) [18]. We suggest that IgG/IgM ratio can eventually be used in some settings/situations as an additional tool for patients’ screening in addition to the traditional clinical warning signs of dengue which still are the best.

One limitation of our study is that we have not differentiated between secondary, tertiary or quaternary dengue. We have primarily differentiated primary from non-primary dengue. We have assumed that the non-primary dengue was secondary dengue in most cases because of the rarity of clinically observed third and fourth infections [29, 30] and also because of the antibodies’ having high cross-reactivity which complicates the distinction of two or more preceding infections.

## Conclusion

We found secondary dengue to cause a more severe disease than primary making the differentiation important. Early on IgG/ IgM ratio can play an important role to differentiate the two. We found the ratio of ≥ 1.10 on day 2 of illness to be the best cut off for the same.

## Abbreviations

DENV: Dengue viruses
DF: Dengue fever
DHF: Dengue haemorrhagic fever
DSS: Dengue shock syndrome
ELISA: Enzyme-linked immunosorbent assays
HI: Hemagglutination inhibition
ROC: Receiver operator curve
SPSS: Statistical Package for the Social Sciences
WHO: World Health Organization
